# A unifying computational framework for stability and flexibility of arousal

**DOI:** 10.3389/fnsys.2014.00192

**Published:** 2014-10-20

**Authors:** Christin Kosse, Denis Burdakov

**Affiliations:** ^1^Neurophysiology, MRC National Institute for Medical ResearchLondon, UK; ^2^MRC Centre for Developmental Neurobiology, King’s College LondonLondon, UK

**Keywords:** arousal, hypothalamus, narcolepsy, orexin, hypocretin, histamine, computation, control

## Abstract

Arousal and consciousness flexibly adjust to salient cues, but remain stable despite noise and disturbance. Diverse, highly interconnected neural networks govern the underlying transitions of behavioral state; these networks are robust but very complex. Frameworks from systems engineering provide powerful tools for understanding functional logic behind component complexity. From a general systems viewpoint, a minimum of three communicating control modules may enable flexibility and stability to coexist. Comparators would subtract current arousal from desired arousal, producing an error signal. Regulators would compute control signals from this error. Generators would convert control signals into arousal, which is fed back to comparators, to make the system noise-proof through self-correction. Can specific neurons correspond to these control elements? To explore this, here we consider the brain-wide orexin/hypocretin network, which is experimentally established to be vital for flexible and stable arousal. We discuss whether orexin neurons may act as comparators, and their target neurons as regulators and generators. Experiments are proposed for testing such predictions, based on computational simulations showing that comparators, regulators, and generators have distinct temporal signatures of activity. If some regulators integrate orexin-communicated errors, robust arousal control may be achieved via integral feedback (a basic engineering strategy for tracking a set-point despite noise). An integral feedback view also suggests functional roles for specific molecular aspects, such as differing life-spans of orexin peptides. The proposed framework offers a unifying logic for molecular, cellular, and network details of arousal systems, and provides insight into behavioral state transitions, complex behavior, and bases for disease.

## Introduction

In many everyday situations, it is vital for arousal and consciousness to remain stable despite changes in mental state. Failure of such arousal “cruise-control” can produce deleteriously unstable arousal, as seen in narcoleptic patients who lose consciousness during emotional disturbances such as laughter. On the other hand, it is also important for the arousal drive to be flexible and modifiable by salient features of the environment, such as metabolic needs or the time of day. This presumably evolved because the energy cost of arousal (Dworak et al., 2010) makes it more economical to tune arousal to demand, rather than switch it on and off in a binary, flip-flop manner. Failure of arousal to be flexible can lead to debilitating disorders such as insomnia. The subject of this article is to explore the currently unclear mechanisms through which brain arousal systems may combine stability/robustness with flexibility.

We shall present a systems perspective on this subject. To define our terms, consciousness and arousal will be used to simply refer to brain states distinct from sleep/unconsciousness. By systems perspective, we mean a level of description lying between generalized mathematics and specialized jargons of neuroscience subfields. Systems views help understanding, because translating a specific problem (e.g., a molecularly defined disease) into a more general “systems” language makes it understandable to other fields and thus addressable by more tools than typically available in one specialized field (Wiener, 1965; Csete and Doyle, 2002, 2004; Doyle and Csete, 2011; von Bertalanffy, 2013). Many specific health problems today are linked to the hypothalamus (obesity, sleep disorders, e.g., Mignot et al., 2002; Yeo and Heisler, 2012; Horvath et al., 2014). Despite huge advances in specialized knowledge (Sohn et al., 2013), these problems remain unsolved and might benefit from additional tools.

From classic clinical studies to modern genetic work, increasingly detailed evidence emerged that the lateral hypothalamus provides a critical part of the brain hardware required for stable consciousness. Almost a century ago, based on post-mortem studies of brains of patients with “sleeping sickness”, von Economo suggested that the lateral hypothalamus is critical for the control of wakefulness and arousal (Saper et al., 2001). Around 70 years later, the lateral hypothalamus was discovered to be the unique location of neurons containing neuropeptide transmitters orexins/hypocretins (de Lecea et al., 1998; Sakurai et al., 1998). Loss of orexin neurons, orexin type-2 (OXR2) receptors, or orexin peptides leads to the narcoleptic inability to maintain stable wakefulness (Sakurai, 2007). Most cases of human narcolepsy, a relatively frequent neurological disorder (1:2000 people, Nishino and Kanbayashi, 2005), are thought to be due to loss of orexin neurons (Nishino et al., 2000; Thannickal et al., 2000; Dauvilliers et al., 2007). Without orexin neurons, arousal can still be generated in normal amounts, but it is neither flexible nor robust (see Section “Are Orexin Neurons Comparators?” below). Orexin neurons are now seen as vital orchestrators of other arousal systems distributed throughout the brain, such as the aminergic systems (see Section “A Modular Model for Neural Control of Arousal in An Uncertain World” below).

From their cell bodies in the lateral hypothalamus, orexin neurons project to almost the entire brain, and their projections largely mirror the distribution of OXR1 and OXR2, the two G-protein coupled receptors for orexin (Peyron et al., 1998; Sakurai, 2007). The firing of orexin neurons stimulates awakening (Adamantidis et al., 2007), and the classic arousal neurons of the brain are innervated by orexin fibers and excited by orexin peptides (histamine, serotonin, noradrenaline neurons, Sakurai, 2007).

These and other experimental studies of orexin networks (reviewed in de Lecea et al., 2006; Sakurai, 2007) define brain hardware required for robust-yet-flexible arousal. However, how these components are arranged to implement wakefulness-stabilizing mechanisms is still unclear. In this review, we consider how experimentally-discovered components can be generalized to operations, and how this may improve understanding of arousal control, including diagnosis and arousal malfunction in sleep disorders. We argue that in the real world, arousal control would be inaccurate if brain arousal generators varied their activity in simple proportion to the environment-controlled activity of orexin neurons. We discuss how different neurons and their connections may implement better mechanisms (e.g., integration, feedback) that are needed for robust-yet-flexible arousal. We produce experimental predictions for testing which neurons implement which aspects of control, and conclude with limitations of the presented models. In broader terms, this article considers a conceptual unification of specific biology of stable wakefulness and general mechanisms for stability as studied in non-biological sciences (e.g., in fields variously known as cybernetics, general systems theory, and control theory, Wiener, 1965; Csete and Doyle, 2002; von Bertalanffy, 2013).

## Circuit motifs for stable-yet-flexible control

**Figure 1:**
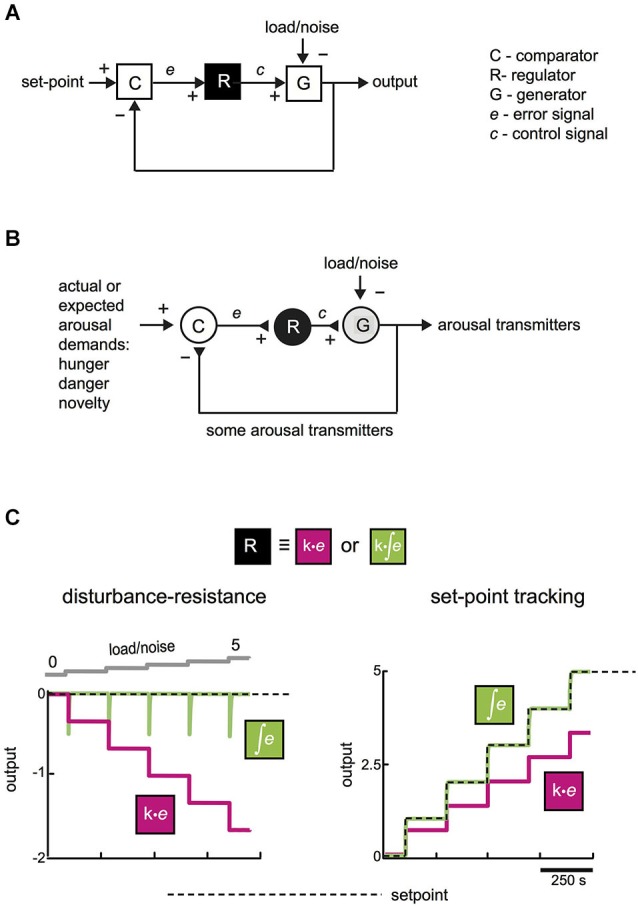
**Functional connectivity for making a stable-yet-flexible signal. (A)** A general mechanism (feedback loop) for tracking a set-point despite disturbance (after Csete and Doyle, 2002). For simplicity, load/noise is modeled as entering at a final summing point, but it can enter the system at any point. **(B)** An equivalent representation of a neural circuit for producing appropriate levels of arousal-enhancing neurotransmitters. **(C)** System performance with and without integral control. Computational simulations of temporal dynamics of the system shown in **(A)**, in the face of changes in load/noise or changes in desired set-point. Left, effect of escalating disturbance on system output with (green) and without (magenta) integration in the module R (*k* = 1 in both cases). Right, response of system output to changes in desired set-point for the two types of control. Integration here had a time window longer than the simulation time; effects of varying the integration window are described in the text and Figure 2A. See Supplementary Figure for more information on computational simulations.

From a general systems point of view, it can be argued that pressure to develop good policies for stable-yet-flexible function is not unique to biological evolution (Csete and Doyle, 2002, 2004). For example, under consumer pressure, in recent decades diverse robotic devices (thermostats, car cruise-controllers) also evolved remarkably effective strategies to make their controlled variables (temperature, speed, etc.) follow set-points despite disturbance. For example, a car cruise-controller can defend set-point speed despite unpredictable variations in slope, wind, or component function, by employing a feedback mechanism (Figure 1A). It has been proposed that, faced with similar general problems (i.e., tracking a set-point despite unpredictable noise), biological and engineering systems undergo a convergent evolution and arrive at similar solutions, such as self-correction via feedback (Csete and Doyle, 2002, 2004). Reverse engineering of biology may therefore benefit from broader frameworks in engineering (Csete and Doyle, 2002; Franklin and Wolpert, 2011).

**Figure 2:**
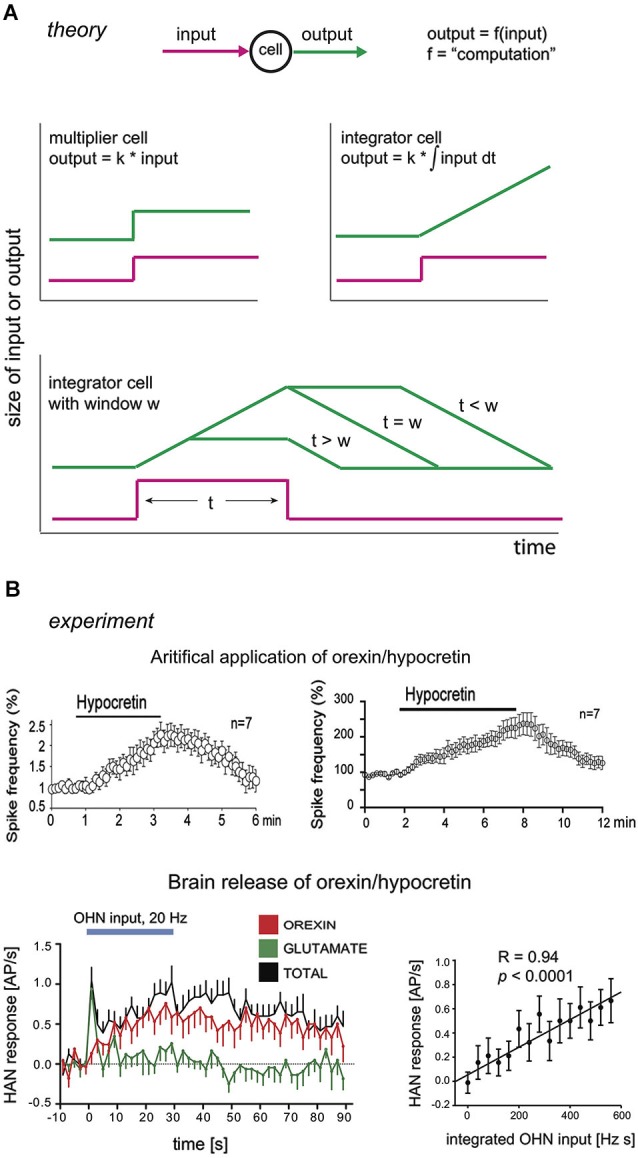
**Integrators and their hypothetical neural correlates. (A)** Theoretical input-output dynamics of neurons that act as multipliers (top left graph), or integrators (top right graph) with different integration windows (bottom graph). W here is defined as the duration of integration window, i.e., given $\int_{b}^{a}input\;dt,w=a-b$. Input is shown in magenta, and output in green. **(B)** Experimental input-output relations of neurons in brain orexin circuits. Top row, firing outputs of orexin/hypocretin neurons (OHNs ) in response to bath application of 1 µM (left) and 200 nM (right) orexin/hypocretin peptides (from Li and van den Pol, 2006, reproduced with permission from The Society for Neuroscience). Bottom row, firing outputs of histamine neurons (HAN) in response to optogenetic stimulation of OHNs at 20 Hz (blue bar). Responses due to orexin transmission are in red (data from Schöne et al., 2014).

**Figure 3:**
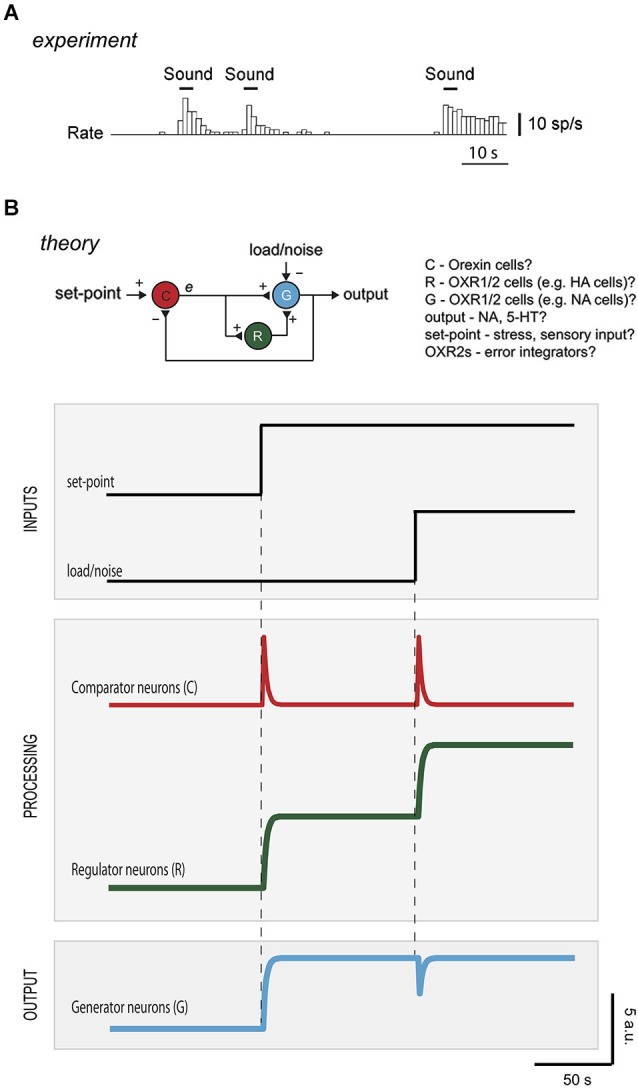
**Diagnosing control roles of neurons from temporal activity pattern. (A)** Firing of an orexin neuron in the hypothalamus of an awake rat experiencing sensory inputs (reproduced with permission from Cell Press from Mileykovskiy et al., 2005). **(B)** Top drawing, hypothetical mapping of control elements to neuronal types in brain orexin circuits. To protect the output (arousal drive) from instability, R could correspond to OXR2-expressing cells (e.g., histamine neurons), and e could come from orexin neurons driven by positive inputs (e.g., sounds, Mileykovskiy et al., 2005) and negative feedback inputs (e.g., serotonin and/or noradrenaline, Li et al., 2002; Yamanaka et al., 2003b). G could correspond to noradrenaline cells, that express OXR1 and are expected to be excited by HANs (Haas et al., 2008). The intermittent firing of orexin cells during wakefulness *in vivo* (Mileykovskiy et al., 2005) is broadly consistent with this placement in the feedback loop. Bottom plots, computational predictions of temporal variations in activity of the different neurons to changes in set-point or in load/noise (R is modeled as an integrator with a time window that exceeded simulation duration; C and G are modeled as algebraic summing points of the inputs they receive, DiStefano et al., 2012). See Supplementary Figure for more information on computational simulations.

From considerations of convergent evolution, appropriate arousal in an uncertain world may also require a feedback mechanism. In its simplest form, this may be implemented by three elements: comparator (C), regulator (R), and generator (G), which achieve appropriate output via a feedback loop (Figure 1B). The comparator computes an error signal, which tells the regulator how much generator output is deviating from desired performance. The regulator is useful, because it can compensate for disturbance without affecting the final (generator) output of the system (see simulation in Figure 3B). There has been debate in some hypothalamus-related literature about set-points vs. settling points (Speakman et al., 2011); in our model the set-point is variable and can be viewed as a settling-point of various arousal demands listed in Figure 1B (but we use the term set-point for brevity).

Theoretical and practical studies suggest that, for tracking despite noise, a particularly effective signal that regulators can send to generators is based on temporal integration of the error signal (integral feedback, Aström and Hagglund, 1995; Yi et al., 2000; Csete and Doyle, 2002; DiStefano et al., 2012). In qualitative terms, error integration enables perfect control based on an accumulated history (“past”), rather than the instantaneous value (“present”), of errors. The quantitative advantage of error integration for precision-tracking of a set-point is demonstrated by a simple simulation in Figure 1C. Control speed (but not the precision of keeping to a set-point) can also be improved by adding regulator actions based on “future”, i.e., time derivative, of error (Aström and Hagglund, 1995; DiStefano et al., 2012). For example, glutamate released by orexin cells can create derivative-like control signals (Figure 2B; see Schöne et al., 2014), but unlike orexin release considered below, glutamate release is insufficient for consciousness stability (Chemelli et al., 1999).

If convergent evolution exists, considerations of general models for control (Figure 1A) may illuminate the function of different types of neurons underlying stable-yet-flexible arousal. In Section “A Modular Model for Neural Control of Arousal in An Uncertain World”, we discuss literature suggesting that some elements of brain orexin circuits are similar to control schemes in Figures 1A,B. In Section “Model Predictions”, we use computational simulations to generate experimentally-testable predictions to evaluate whether specific neurons may indeed behave as specific control modules.

## A modular model for neural control of arousal in an uncertain world

We note that brain arousal-control systems resemble the schemes in Figures 1A,B in some general respects:

1. Distinct modules are involved, including classic arousal-controling clusters (noradrenaline, histamine, serotonin neurons) and more recently discovered clusters (orexin neurons).
2. These modules are non-redundant (if the few thousands of orexin cells are missing, remaining billions of neurons cannot compensate (Hara et al., 2001). This argues for serial, non-redundant control loops, rather than grossly redundant parallel networks.
3. There is evidence for feedback signals to orexin cells from “arousal generator” chemicals such as some aminergic transmitters (see Section Are Orexin Neurons Comparators?).

The canonical feedback loop (Figure 1A) may therefore be implemented in the brain by specific neurons that are wired together and controlled by physiologically relevant inputs (Figures 1B, 3B). In this context, load/noise (Figures 1A,B, 3B) would correspond to anything that opposes the function of the arousal neurons (e.g., a spontaneous inhibitory input from sleep neurons). A definite mapping of cell types to control modules (C-R-G, Figure 3B) needs more experimental tests, as suggested in section “Model Predictions”. However, existing data raise the following questions:

### Are orexin neurons comparators?

Studies of narcolepsy (in dogs, mice, humans) caused by loss of orexin neurons, type-2 receptors, or orexin peptides, illustrate that orexin cells are vital for arousal cruise-control (Chemelli et al., 1999; Lin et al., 1999; Peyron et al., 2000; Willie et al., 2003). First, orexin neurons and receptors are essential for protecting consciousness from instability during emotional excitement (Dauvilliers et al., 2007). In orexin-deficient brains, emotional load, e.g., excitement from a funny joke, winning a game, playing, or exposure to a favorite food, can inappropriately reduce normal wakefulness and produce sleep attacks (Dauvilliers et al., 2007). Second, the orexin system ensures that arousal levels track environmentally-relevant set-points. Knockout of orexin peptides greatly reduces normal arousal responses (such as elevated locomotion, heart rate, breathing) to food shortage, high CO_2_, or stress (Yamanaka et al., 2003a; Sakurai, 2007; Kuwaki, 2011). However, orexin neurons are not essential for wakefulness *per se*: their loss fragments wakefulness but does not change its total amount per day (Hara et al., 2001). This suggests that orexin neurons are not themselves generators of wakefulness (if this were the case, animals lacking orexin would sleep more). Indeed, during wakefulness *in vivo*, orexin cells are not constantly firing, but become stimulated by signals that demand increased wakefulness, such as sudden sounds, stress, hypoglycaemia or CO_2_ (Figure 3A; Yamanaka et al., 2003a; Mileykovskiy et al., 2005; Sakurai, 2007; Williams et al., 2007).

We believe that several experiments are consistent with a comparator (error-generator) role for orexin neurons in wake-control circuitry (module C in Figure 1). *In vitro*, orexin neurons appear to be excited by signals indicative of wakefulness demand (higher set-point), and inhibited by some of the signals indicative of current wakefulness drive. The excitatory signals include CO_2_, unexpected noises, low glucose, stress; and the inhibitory signals include serotonin and, under some circumstances, noradrenaline (Sakurai et al., 1998; Liu et al., 2002; Winsky-Sommerer et al., 2004; Grivel et al., 2005; Mileykovskiy et al., 2005; Sakurai, 2007; Williams et al., 2007, 2008). Noradrenaline effects on orexin cells are currently a subject of discussion: some groups report excitation and propose noradrenaline-induced inhibition to occur only after sleep deprivation (Bayer et al., 2005; Grivel et al., 2005; Uschakov et al., 2011), whereas other groups report only inhibition (Li et al., 2002; Yamanaka et al., 2003b; Williams et al., 2011). In any case, it is still unclear how these *in vitro* studies reflect the effects of noradrenaline cell firing *in vivo*, since orexin-projecting noradrenaline neurons may co-release fast transmitter such as glutamate or GABA (as recently reported for different subpopulations of dopamine neurons, Tecuapetla et al., 2010; Tritsch et al., 2012).

Thus at least some aspect of orexin cell activity may reflect what would be considered an (arousal) error signal in control engineering, since they sum/compare some positive inputs requiring arousal (e.g., sounds) and at least some negative feedback signals of current arousal networks activity (e.g., serotonin). This suggests that orexin neurons might be comparators, but we also note that orexin neurons themselves express functional OXR2s, and are excited by orexin (Figure 2B; see Li and van den Pol, 2006; Yamanaka et al., 2010). Although we do not consider it here, this may mean that some orexin cells (or a subpopulation of them) act as regulators as well as comparators.

### Are OXR2-expression neurons regulators?

In the generic control loop for stability (Figure 1), comparators send error signals to regulators. May such regulators correspond to some of the neurons expressing orexin type-2 receptors (OXR2)? Like orexin peptides (or orexin neurons), the OXR2 cells are essential for wakefulness stability, since OXR2 knockout (but not OXR1 knockout) causes a severe narcoleptic phenotype (Lin et al., 1999; Willie et al., 2003; Mieda et al., 2011). However, like orexin knockout, the OXR2 knockout does not significantly change the total amount of wakefulness (Willie et al., 2003). This seems to imply that, like orexin neurons, OXR2s are not themselves wakefulness generators. Could OXR2 neurons (e.g., histamine cells) be regulators, which receive inputs from comparators and send outputs to wakefulness generators such as noradrenaline neurons (Haas et al., 2008)?

Intriguingly, relations between inputs and outputs (i.e., computations, Figure 2A) of some OXR2 neurons resemble those of regulators in integral control loops (Figure 2B; Li and van den Pol, 2006; Schöne et al., 2014). As discussed above, an integral control loop may be necessary and sufficient for the type of robust flexibility that orexins give to arousal. In an integral feedback loop, the regulator acts as an integrator (Csete and Doyle, 2002; DiStefano et al., 2012). This means that it has a linear relation between its output and the integral of its input, i.e., its response increases during steady input (Figure 2A). Experimentally, when a constant firing input from orexin neurons is optogenetically replayed to histamine neurons (HANs; which express OXR2 and are a key anti-narcoleptic target of orexin cells, Yamanaka et al., 2002; Mochizuki et al., 2011), they increase their firing rate linearly for at least 30 s (Figure 2B; Schöne et al., 2014). In turn, when constant concentrations of orexin are applied by bath to orexin neurons (which express OXR2, Yamanaka et al., 2010), they also keep increasing their firing rate linearly, for at least 6 min (Figure 2B; Li and van den Pol, 2006). Although these data are consistent with OXR2s acting as integrators, they do not yet rule out that OXR2s instead act as low-pass filters, whose input-output relations can be similar to those of integrators. While placing a low-pass filter in the regulator position may achieve reasonable feedback control, such a regulator may require considerably more gain (i.e., be more expensive to implement) than integral feedback.

Some OXR2-expressing neurons may therefore integrate orexin cell input at time scales relevant for processing the durations of orexin cell firing bursts seen *in vivo* (reported to last from seconds to minutes, Mileykovskiy et al., 2005). The computational outcome of this integration will be passed on to downstream arousal generators. We note that, *in vivo* in the brain, orexin peptides will be subject to diffusion/degradation and so postsynaptic effects of neurotransmission may saturate during constant firing of orexin axons. This is likely to constrain integration to a finite time window (i.e., the firing rate of an OXR2 cell may no longer be affected by orexin release from a day ago). Experimentally measurable consequences of this are illustrated in Figure 2A (bottom). A neuron integrating an orexin signal will give different temporal patterns of spiking, depending on how long the input is relative to how fast orexin is removed from receptors (by degradation/diffusion) (Figure 2A). It should therefore be possible to estimate the integration window by experimentally varying input duration (e.g., time for which orexin is applied) and analyzing output dynamics (e.g., temporal variation in OXR2 cell firing). In Section “Model Predictions” below, we speculate that by varying half-lives of orexins (e.g., by adjusting the relative proportions of orexin-A and orexin-B) it may theoretically be possible to tune the integration window for optimal control responses.

### Generators of arousal drive

Generator neurons for arousal would have the following operational features: (1) their activity should promote arousal; (2) their activity would be essential for the signal from comparators (or regulators) to be converted into wakefulness/arousal; (3) their direct modulation by comparators would be insufficient for appropriate arousal; and (4) some aspects of their activity would be sent to comparators as negative feedback. At present, at least some of these properties seem to be exhibited by noradrelanine neurons in the brain’s locus coeruleus. Their activity promotes arousal (Carter et al., 2010). Their activity is essential for the signals from orexin neurons to produce awakening (Carter et al., 2012). Their direct modulation by orexin cell inputs, via OXR1 expressed by noradrenaline neurons (Hagan et al., 1999; Sakurai, 2007), cannot rescue unstable arousal caused by loss of OXR2 from other neural modules (Lin et al., 1999; Willie et al., 2003). Finally, noradrenaline has been shown to inhibit orexin neurons (Li et al., 2002; Yamanaka et al., 2003b), although this effect could change as a function of prior sleep history (Grivel et al., 2005). However, negative feedback to orexin neurons can be mediated by other chemicals indicative of arousal drive, in particular serotonin which robustly inhibits orexin neurons via 5-HT_1A_ receptors (Muraki et al., 2004).

In summary, although specific details remain to be elucidated, we suggest that the above experimental data could be interpreted as consistent with control of arousal by non-redundant modules similar to those in Figure 1A. However, based on current evidence, it cannot be excluded that real control circuits also contain redundant wiring (e.g., parallel connections from several comparator and regulator neurons to several generators).

## Model predictions

A major advantage of expressing theories as formal control diagrams (Figure 1A) is that they can be analyzed computationally, to make quantitative experimental predictions to test the theories. Figure 3B shows computational predictions for temporal activity patterns of different neurons (comparator, regulator, generator). The circuit diagram in Figure 3B has been altered slightly from the basic control scheme in Figure 1A: comparator C now communicates directly with both regulator R and generator G, Figure 3B. This alteration is not critical for conclusions presented here, and was introduced to make the circuit more similar to orexin circuit, in which orexin cells synapse with both putative regulators (e.g., histamine cells) and putative generators (e.g., noradrenaline cells) (Sakurai, 2007).

In response to a step change in set-point, the activities of the interconnected neurons C-G-R rearrange to make the output of G exactly match the new set-point (Figure 3B). Note that temporal response of neuron C (i.e., error signal) is transient, in contrast to sustained responses of neurons R and G, since the system is wired to eliminate the error.

While responses of regulator and generator to a change in set-point are broadly similar (Figure 3B), they can be operationally differentiated by looking at their responses to load/noise (i.e., the type of input that the system is designed to resist). Introduction of a sustained load/noise causes the regulator neurons to produce a sustained change in activity which opposes the load, while the generator neuron activity is unsettled only transiently and then goes back to set-point (Figure 3A). This illustrates why both regulator and generator are important for robust performance. The regulator deals with unwanted signals, so that the generator is not distracted from delivering a set-point.

Experimental activity signatures of different neurons may thus point to their specific control roles and mutual interconnections in a network. Neurophysiological correlates of brain’s set-points remain elusive, but selective manipulations of specific hypothalamic neurons have effects that can be interpreted as changes of vital set-points (Gropp et al., 2005; Luquet et al., 2005; Aponte et al., 2011; Krashes et al., 2011). A load/noise can also be introduced into arousal circuits, e.g., by activating opposing sleep circuits. It should thus be experimentally possible to impose changes in set-points or loads on the brain, to measure consequent responses of different cells, and to compare them with predictions associated with different control architectures and computations, such as those shown in Figure 3B. At present, such investigations are complicated by the lack of universal agreement of how to measure the final output of the system, the level of arousal. For example, the level of arousal can be measured as cortical gamma activity (EEG), and/or as muscle tone and movement (EMG), and/or as sympathetic parameters such as heart rate, blood pressure, and respiration. Choice of most appropriate measure of the level of arousal may ultimately improve our understanding of activity in different arousal-related neurons in relation to EEG, EMG, and sympathetic measures.

Do existing *in vivo* recordings from orexin, histamine, and noradrenaline networks correspond to the predictions in Figure 3B? From the limited number of such recordings that exist in the literature, it is difficult to infer this definitively, without precisely controlled and quantified inputs the system, e.g., introducing a constant level of disturbance such as that shown in Figure 3B (Aston-Jones and Bloom, 1981; Lee et al., 2005; Takahashi et al., 2006). The interpretation of existing data in the context of our model is thus unclear, but there is some evidence that orexin neurons fire transiently in response to disturbances (Mileykovskiy et al., 2005), while histamine and noradrenaline neurons discharge in a tonic fashion (Aston-Jones and Bloom, 1981; Takahashi et al., 2006). This issue needs to be revisited with more quantified experimental stimuli, and with recordings from a greater number of neurons to assess the net output of the networks.

A special note is warranted about the time window integrated by the regulator neurons. As mentioned above, this may correspond to physiological variables such as the active life-span of neurotransmitter molecules (i.e., diffusion/degradation/half-life). A short life-span would mean a brief integration window, and vice versa. A full computational treatment is beyond the scope of this article, but because the integration time window determines the regulator response (Figure 2A), it also determines how quickly the integral feedback circuit deals with changes in set-point or noise. If the life-span of a transmitter is overly short, the circuit will behave like a proportional controller, giving rise to problems illustrated in Figure 1C. If the life-span is overly long, a control signal will persist much longer than the error signal, causing the generator to deviate from set-point for unacceptable amounts of time. Ideally the life-span would thus be tuned to typical durations of inputs (set-point or noise) experienced by the system in real life. Physiologically, for arousal systems these input durations would correspond to durations of salient signals (e.g., 10 s of crossing a busy street) and random disturbances (e.g., 5 min of laughter). Typical durations of these features would obviously vary vastly depending on the environment. It is tempting to speculate that the environment might tune the half-life of the mix of orexin-A and -B released by orexin neurons, in order to optimize the integration window and control. Orexin-A and -B are equally potent activators of the anti-narcoleptic OXR2s, but two disulfide bonds in orexin-A would presumably make it much less easily degradable than orexin-B (Sakurai et al., 1998). If the brain could vary the proportions of orexin-A and -B released onto OXR2 neurons, we predict that an environment filled with rapidly changing inputs should increase orexin-B/orexin-A ratio (e.g., by environment-dependent processing of the pre-proorexin precursor peptide). This would result in a shorter integration window and better tracking of error signals by the control system (Figure 2A).

## Conclusions and limitations

We have presented a systems framework that unifies biologically-plausible neurophysiology and appropriate cruise-control of arousal. This framework highlights the need for at least three types of neurons with specific connections and response properties. Several types of arousal-promoting neurons have been discovered in the brain (Saper et al., 2001; Jones, 2003). We suggest that these distinct neurons may implement distinct, non-redundant algorithms (Figure 1A). The proposed framework also highlights weak-points in the system. If an irrelevant input/noise enters the system at the level of regulator or generator, it may be reasonably counteracted by the feedback loop so that the generator output remains appropriate (as in Figure 2B). However, if disruption enters at the level of the comparator, the consequences could be disastrous, since the system would process it as a salient set-point (i.e., generator output will be disrupted). These considerations are useful for at least two reasons. First, for interpreting experiments aimed at identifying roles of neurons (comparator, regulator, generator) in behavior, e.g., during targeted optogenetic interventions combined with behavioral readouts (Adamantidis et al., 2010; Atasoy et al., 2012). Second, for understanding why disrupting a tiny fraction of certain brain cells (e.g., few thousand orexin neurons out of billions of neurons in the brain) can have such dramatic impact on brain state control.

The proposed model is intended as a useful guide for rationalizing the vast complexity of arousal control components in the brain, and for designing new experiments. We acknowledge that it does not explicitly consider many fundamental aspects of brain state control, and has other limitations. For example, aspects such as plasticity (learning and memory) in the cruise-control circuit remain relatively unexplored. We also neglected the potentially intricate dynamics of sleep-promoting signals (Achermann and Borbély, 2003; Fulcher et al., 2014) by viewing such signals as noise during wakefulness. Our model focuses on wakefulness only and does not deal with sleep; other engineering analogies, such as flip-flop control circuits, have been proposed for sleep state control (Lu et al., 2006).

Despite these limitations, the proposed framework highlights the importance of specific computations for arousal control in the real world. Current (drug) therapies for arousal disorders (insomnia, narcolepsy) are essentially aimed at increasing brain concentrations of chemicals that suppress or promote arousal (Saper and Scammell, 2013). Such drug strategies may not replay brain computations required for proper arousal control (Figures 1C, 2B). Indeed, current drug therapies for narcolepsy and insomnia have mild therapeutic success with many unwanted side-effects (Bhat et al., 2008; Saper and Scammell, 2013). An alternative therapy would be to compute and re-introduce appropriate brain activity through electroceuticals and closed-loop control of brain function (Armstrong et al., 2013; Famm et al., 2013). Computational frameworks such as those presented here would be critical for success of such therapies, when tools for targeted closed-loop control of the human brain become sufficiently developed.

## Author contributions

Invited contribution to Frontiers in Systems Neuroscience, on a Research Topic titled “A Systems Approach to Understanding Recent Advances in Hypothalamic Structure and Function”.

## Conflict of interest statement

The authors declare that the research was conducted in the absence of any commercial or financial relationships that could be construed as a potential conflict of interest.
