# Supplementary material for: A unifying computational framework for stability and flexibility of arousal
Source: Front Syst Neurosci. 2014 Oct 20;8:192. doi: 10.3389/fnsys.2014.00192 (PMC4202806; doi:10.3389/fnsys.2014.00192)
Supplement: Supplementary file 1 [file Image_1.PDF]

Supplementary Figure, Kosse and Burdakov

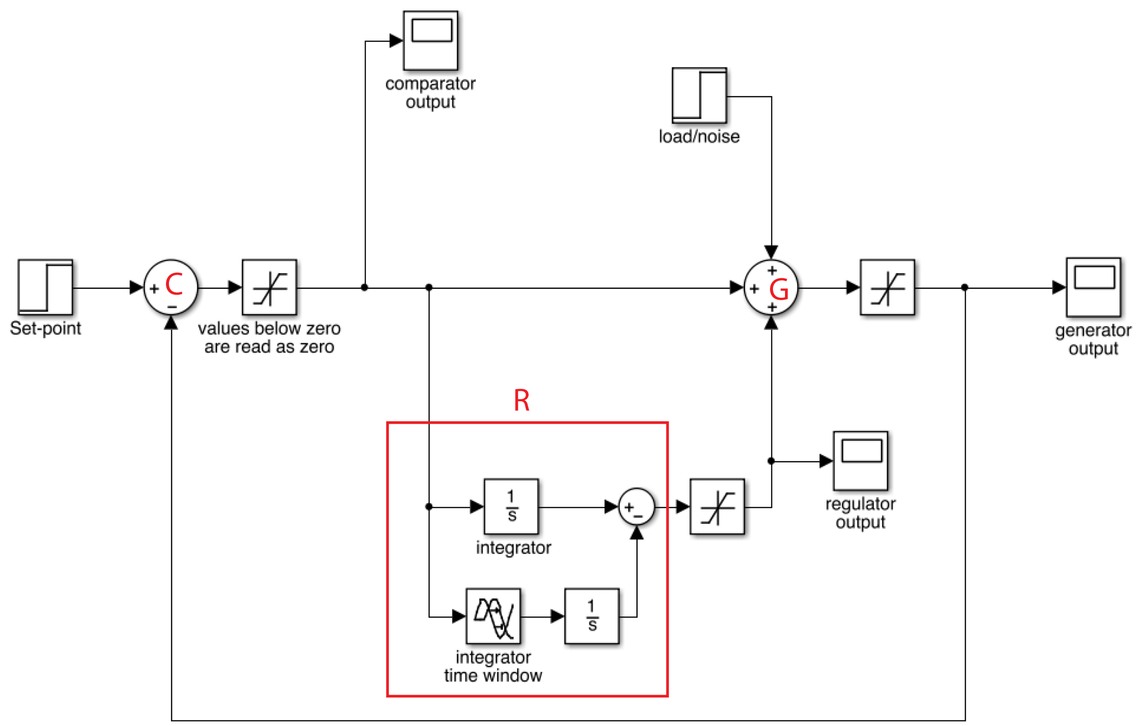

An example of a Matlab-Simulink program used to generate computational simulations.

Signals were constrained to zero or above, since firing rates of real neurons cannot be negative. Red labels are not part of the program but are given for clarify to show the parts corresponding to comparator (C), regulator (R), and generator (G) in the main text.
